# Comparing the difference of adverse events with HER2 inhibitors: a study of the FDA adverse event reporting system (FAERS)

**DOI:** 10.3389/fphar.2024.1288362

**Published:** 2024-01-24

**Authors:** Yiwen Bao, Jiaju Chen, Luting Duan, Fujue Wang, Han Lai, Zeming Mo, Weiliang Zhu

**Affiliations:** ^1^ Department of Oncology, The People’s Hospital of Qiannan, Duyun, Guizhou, China; ^2^ Department of Oncology, Zhujiang Hospital, Southern Medical University, Guangzhou, China; ^3^ Gastrointestinal Surgery, The Affiliated Hospital of Guizhou Medical University, Guiyang, Guizhou, China; ^4^ Department of Cardiovascular Medicine, The People’s Hospital of Qiannan, Duyun, Guizhou, China; ^5^ State Key Laboratory of Biotherapy and Cancer Center, West China Hospital, Sichuan University and Collaborative Innovation Center, Chengdu, China; ^6^ Division of Head and Neck Tumor Multimodality Treatment, Cancer Center, West China Hospital, Sichuan University, Chengdu, China

**Keywords:** HER2 inhibitors, FAERS database, adverse drug events, hemorrhagic events, nervous system disorders, TDM-1, Tucatinib

## Abstract

**Aim and background:** This study attempted to identify similarities and differences in adverse events (AEs) between human epidermal growth factor receptor 2 (HER2) inhibitors, especially those related to hemorrhagic events and nervous system disorders.

**Methods:** This study summarized the types, frequencies, and system organ classes (SOCs) of AEs of HER2 inhibitors. The US Food and Drug Administration Adverse Event Reporting System (FAERS) data from January 2004 through March 2022 was collected and analyzed. Disproportionality analyses were conducted to detect AEs signals for every HER2 inhibitor. The chi-square test, Wilcoxon test, and descriptive analysis were used to compare the differences of AEs for specific SOCs or drugs.

**Results:** A total of 47,899 AE reports were obtained for eight HER2 inhibitors. Trastuzumab-related AEs were reported in the highest number and combination of regimens. In monotherapy, trastuzumab had the highest reported rate of cardiac disorders-related AEs (24.0%). However, small-molecule drugs exceeded other drugs in the reported rates of AEs related to gastrointestinal disorders, metabolism and nutrition disorders. The highest reported rates of respiratory disorders (47.3%) and hematologic disorders (22.4%) were associated with treatment with trastuzumab deruxtecan (T-DXd). Patients treated with trastuzumab emtansine (TDM-1) had the highest reported rate (7.28%) of hemorrhagic events, especially intracranial haemorrhage events. In addition, patients treated with TDM-1 with concomitant thrombocytopenia were likely to experience hemorrhagic events compared to other HER2 inhibitors (*p* < 0.001). The median time to onset of intracranial haemorrhage associated with trastuzumab (0.5 months) and TDM-1 (0.75 months) was short. However, there was no significant difference in median time to onset intracranial haemorrhage between patients in different age groups or with different outcomes. *Disproportionality analysis* results reveal that cerebral haemorrhage is a positive signal associated with T-DXd and TDM-1. In addition, tucatinib was the drug with the highest rate of reported nervous system disorders (31.38%). Memory impairment (83 cases) is a positive signal for tucatinib.

**Conclusion:** The types and reporting rates of AEs associated with different HER2 inhibitors vary across multiple systems. In addition, hemorrhagic events concomitant with TDM-1 treatment and nervous system disorders concomitant with tucatinib treatment may be worthy of attention.

## 1 Introduction

HER2 is a vital driving gene for many malignant tumors, such as breast cancer (BC), gastric cancer, and ovarian cancer. Overexpression and amplification of HER2 are closely related to the rapid progress of tumors (Cianfrocc et al., 2004; Rakhshani et al., 2014; Berchuck et al., 1990). Considering the incidence and prevalence of these tumors worldwide, how to effectively control the invasion and metastasis of HER2-positive (HER2+) tumors has become an important issue. Due to the reliability of HER2 as a target antigen, the types of HER2 inhibitors are diversifying. Currently, there are three main types of HER2 inhibitors used in clinical practice: small-molecule drugs (lapatinib, neratinib, tucatinib, and pyrotinib), monoclonal antibodies (trastuzumab, pertuzumab), and antibody-conjugated drugs (ADCs): TDM-1, T-DXd, et al. (Schlam and Swain, 2021; Singh et al., 2022; Banys-Paluchowski et al., 2023). For example, many guidelines recommend the combination of trastuzumab, pertuzumab, and taxane as the standard first-line treatment for patients with advanced HER2+ BC (Swain et al., 2020). In addition, T-DXd has been successively approved for second-line treatment of BC and gastric carcinoma and also approved for treatment of metastatic BC with low HER2 expression (Grieb and Agarwal, 2021; Singh et al., 2022). In addition, the number of cancer types for which HER2 inhibitors are used is still increasing (Oh and Bang, 2020). Moreover, as the reliability of HER2 inhibitor efficacy has been validated, many oncology patients have received different HER2 inhibitors in concomitant combinations or phases (Giordano et al., 2022).

Many studies have suggested that HER2 inhibitors related to adverse events (AEs) significantly reduce patients’ quality of life, lead to treatment interruption, and ultimately impair the efficacy of inhibitors and the survival period of patients (Hedhli and Russell, 2011; Zambelli et al., 2011). However, there are still relatively few reports on the lateral comparison of AEs in all SOCs. Therefore, we were curious if the AEs in patients treated with only one HER2 inhibitor differed from those who received multiple HER2 inhibitors. Furthermore, are there significant differences in AEs associated with different types of HER2 inhibitors? For example, some studies have found severe hemorrhagic events with treatment with TDM-1 (Thuss-Patience et al., 2017; Delgado et al., 2021; Wuerstlein et al., 2022). The issue of potential hemorrhagic events with the use of other HER2 inhibitors is then equally worth evaluating. Meanwhile, because the permeability of the blood-brain barrier between small-molecule drugs and ADCs is significantly different, the differences in these drug-induced nervous system disorders remain unknown (Stemmler et al., 2007; Guntner et al., 2020). FAERS database can provide a full range of reported events related to AEs of different drugs in real-world evidence from worldwide (Mo et al., 2023). Therefore, this study is based on the FAERS database to assess the differences in multiple SOC-related AEs suspected to be associated with one HER2 inhibitor or multiple HER2 inhibitors, especially those related to hemorrhagic events and nervous system disorders. Subsequently, the reported rates of AEs for preferred terms (PTs) and affiliated SOCs associated with monotherapy and combination therapy were itemized.

## 2 Materials and methods

### 2.1 Data collection and extraction

Data for the retrospective pharmacovigilance study were obtained from the FAERS database. FAERS database is a recognized source for timely, real-world safety assessments of drugs and therapeutic biological products (https://fis.fda.gov/extensions/FPD-QDE-FAERS/FPD-QDE-FAERS.html). FAERS database was searched (1 January 2004–31 March 2022) for AEs data of FDA-approved HER2 inhibitors (lapatinib, neratinib, tucatinib, pyrotinib, trastuzumab, pertuzumab, TDM-1, T-DXd across all indications). Trade names and generic names of drugs in the National Center for Biotechnology Information (NCBI) were used as the search terms for HER2 inhibitors. Since Product Active ingredients (PROD_AI) were added to drug/biologic information (DRUG) of FAERS data files after 2014, PROD_AI of drugs was also added as search terms. Then, the relatively comprehensive results were obtained.

### 2.2 Data cleaning procedures

We only selected reported events for HER2 inhibitors judged to be primary suspect (PS) and second suspect (SS) in “ROLE_COD”. As recommended by the FDA, we removed duplicate records prior to statistical analysis by selecting the most recent FDA_DT when the CASEID was the same and the higher PRIMARYID when the CASEID and FDA_DT were the same. Specific reports indicated as erroneous on the FDA website were removed as recommended. The preferred term (PT) of the Medical Dictionary for Regulatory Activities (MedDRA) was used to standardize the AEs data, and SOC was utilized to classify AEs into various systems. The data cleaning process relied on SAS statistical soft-ware (version 9.4; SAS Institute, Cary, NC, United States). Cases with only a single HER2 inhibitor in the DRUGNAME list were categorized as monotherapy. Cases with two or more HER2 inhibitors in the DRUGNAME list were categorized as combination therapy. Finally, all cases that contained the same PRIMARYID for the combination therapy were finally de-weighted again.

### 2.3 Statistical analysis

#### 2.3.1 Disproportionality analysis.

*Disproportionality analysis* is a data mining method now widely used in monitoring adverse drug reactions (Chuma et al., 2022; Raschi et al., 2022). The Proportional Reporting Ratio (PRR) method, and Bayesian Confidence Propagation Neural Network method were utilized to detect safety signals for the drugs under study. We calculated PRR, information components (IC) and the corresponding 95% confidence intervals lower limit by using a 2 × 2 contingency table to detect potential associations between HER2 inhibitors and AEs. The AEs was considered significantly associated with the targeted drug relative to other drugs when the number of cases of AEs was greater than 3, PRR ≥ 2, and the lower limit of the 95% CI of PRR values exceeded 1.0.

#### 2.3.2 Descriptive analysis

The adverse event reporting rate was set as the total number of specific AEs as a percentage of the total number of cases for the targeted HER2 inhibitor. Hemorrhagic events include AEs related to haemorrhage and hemorrhagic in the pt_name list. The time of onset of an intracranial hemorrhagic event was recognized to be the time of the event minus the time of initial treatment with the drug.

The chi-square test was performed based on the R package ggstatsplot (version 0.12.1; Indrajeet Patil) (Patil, 2021); The Wilcoxon test and the Kruskal Wallis test were performed based on the R package ggpubr (version 0.6.0; Alboukadel Kassambara [aut, cre]) (Kassambara, 2023).

#### 2.3.3 Data visualization

ggplot2 (version 3.4.1; Hadley Wickham) (Wickham, 2016), ggVennDiagram (version 1.4.9; Chun-Hui Gao) (ggVennDiagram, 2023), ggalluvial (version 0.12.5; Hadley Wickham) (Brunson, 2020), ComplexHeatmap (version 2.16.0; Zuguang Gu) (Gu et al., 2016), and UpSetR (version 1.4.0, Jake Conway, Nils Gehlenborg) (Conway et al., 2017) were employed for visualization. Data visualizations were processed by the software Rstudio (version 2023.03.0; Build 353 ^©^ 2009–2022 Posit Software PBC) in the R environment (version 4.3.2). *p* < 0.05 was statistically significant, and the *p*-value was bilateral.

## 3 Results

### 3.1 Basic clinical characteristics of patients treated with HER2 inhibitors

**FIGURE 1:**
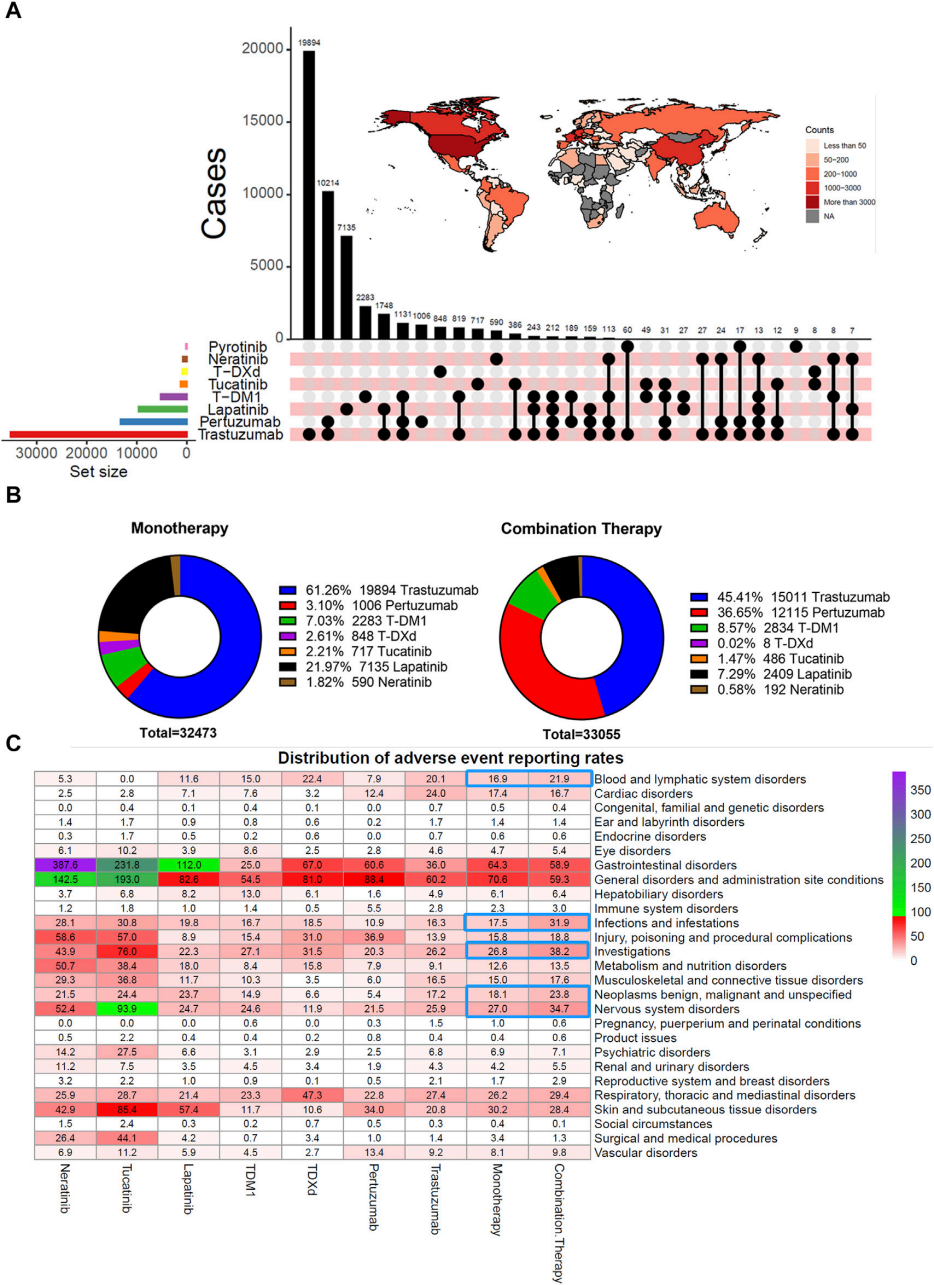
Clinical characteristics of patients treated with HER2 inhibitors and distribution of adverse event reporting rates. **(A)** Visualization of overlapping relationships between users of different HER2 inhibitors and the total number of all HER2 inhibitors reported in different countries was based on the R packages UpSetR and ggplot2. **(B)** Number and percentage of cases of each HER2 inhibitor in monotherapy and combination therapy. Considering that the total number of pyrotinib was too small, they were excluded from monotherapy and combination therapy statistics. **(C)** Heat map displays the distribution of adverse event reporting rates for seven HER2 inhibitors.

What are the clinical characteristics of patients treated with HER2 inhibitors in reports derived from the FAERS database? After extracting and cleaning the FAERS raw data, all subtypes of the final eight HER2 inhibitors were identified for monotherapy and combination therapy (Figure 1A). Among monotherapy, trastuzumab (19,894 cases), lapatinib (7,135 cases), TDM-1 (2,283 cases), and pertuzumab (1,006 cases) had the top 4 highest number of reported cases. Trastuzumab plus pertuzumab (10,214 cases), trastuzumab plus lapatinib (1,748 cases), trastuzumab plus pertuzumab plus TDM-1 (1,131 cases), and trastuzumab plus TDM-1 (819 cases) are the first four common HER2 inhibitor combination strategies. In addition, we found that some patients received a combination strategy of three, four, or even five HER2 inhibitors. Figure 1A also shows the total number of reported HER2 inhibitor-related AEs in different countries worldwide. The United States (9,281 cases), United Kingdom (3,190 cases), Japan (2,687 cases), and China (2,629 cases) were the top 4 countries in terms of the number of AEs. In addition, the number of reported cases of pertuzumab in combination therapy was significantly higher (12,115 cases) than in monotherapy (1,006 cases) (Figure 1B).

In monotherapy, trastuzumab had the highest reported rate of cardiac disorders-related AEs (24.0%) (Figure 1C). However, small molecule agents exceeded monoclonal antibodies and ADCs in the reported rates related to gastrointestinal disorders, metabolism and nutrition disorders, and skin and subcutaneous tissue disorders. The reported rate of gastrointestinal disorders was more pronounced in patients who had received neratinib. The highest reported rates of respiratory disorders (47.3%) and hematologic disorders (22.4%) were associated with treatment with T-DXd. Patients treated with TDM-1 had the highest reported rate (7.28%) of hepatobiliary disorders. These results suggest differences in the presence of potential AEs for each HER2 inhibitor. In addition, combination therapy had a higher reported rate of AEs involving blood and lymphatic system disorders, infections and infestations, investigations, neoplasms benign, malignant and unspecified, and nervous system disorders than monotherapy.

### 3.2 Distribution statistics of adverse events based on PTs and SOCs classification in monotherapy

**FIGURE 2:**
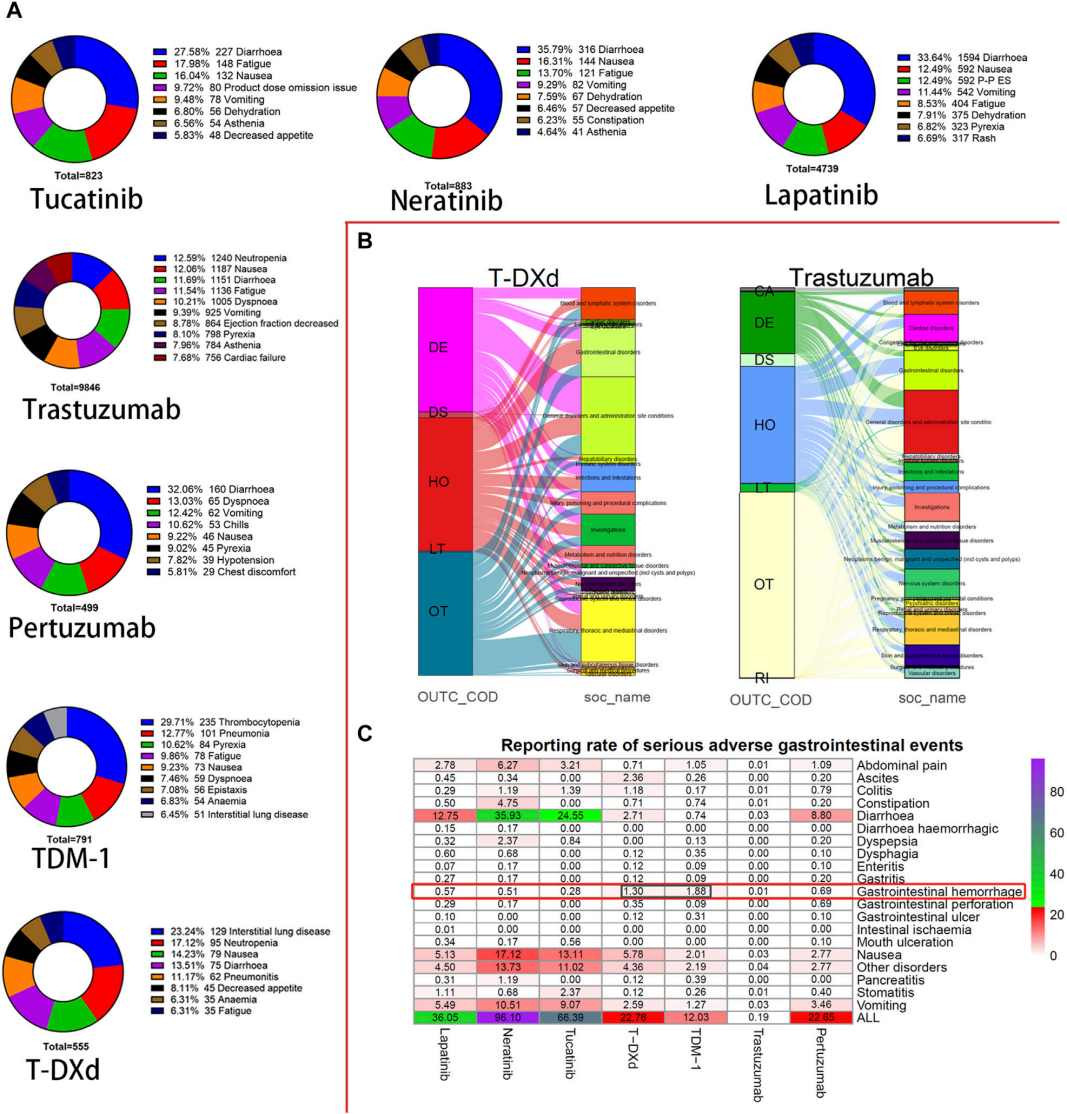
Distribution statistics of adverse events (AEs) based on PTs’ and SOCs’ classification in monotherapy. **(A)** Pie chart visualizing the number of AEs reported at high frequency for the seven HER2 inhibitors. **(B)** Mulberry diagram visualizing the correspondence of adverse events (soc_name) and outcomes (OUTC_COD) reported in cases treated with T-DXd and trastuzumab. **(C)** Heat map displays the distribution of AEs reporting rates of gastrointestinal disorders for seven HER2 inhibitors.

The frequency and type of PTs that were primarily suspected and secondarily suspected of being associated with monotherapy were counted (Figure 2A). Diarrhea was the most frequently reported PTs for tucatinib, neratinib, lapatinib, and pertuzumab. Besides, fatigue, nausea, and vomiting were also common AEs in all HER2 inhibitor users. In addition, dehydration is a common PTs for small-molecule drugs. However, ejection fraction decreased (8.78%) and cardiac failure (7.68%) were the top 10 reported PTs for trastuzumab application, which was not found for other drugs. Another concern is thrombocytopenia (235 cases), the most frequently reported AEs in TDM-1 applicants. Pneumonia (101 cases) occurrence is a close second. Pulmonary and hematologic toxicity remains a significant concern for TDM-1 applications.

Subsequently, the inter-correlation between the outcomes of AEs and SOCs of HER2 inhibitor monotherapy was visualized (Figure 2B, Supplementary Figure S1). We found that gastrointestinal disorders accounted for a relatively high proportion of specific adverse severe outcomes (death, life-threatening, and hospitalization—initial or prolonged) for all HER2 inhibitors. The distribution of gastrointestinal disorders among patients with specific serious adverse outcomes was further analyzed, and the reporting rate of AEs was counted. Diarrhea, nausea, vomiting, and abdominal pain were the higher reported PTs, with neratinib- and tucatinib-related ones being more pronounced (Figure 2C). At the same time, trastuzumab had a very low reporting rate for almost all PTs. However, the reported rates of T-DXd (1.30%) or TDM-1 (1.88%) associated gastrointestinal haemorrhage were greater than 1%, significantly exceeding that of other HER2 inhibitors. Therefore, we pondered whether there was a pharmacologic predisposition to the occurrence of hemorrhagic events.

### 3.3 Hemorrhagic events with HER2 inhibitors

**FIGURE 3:**
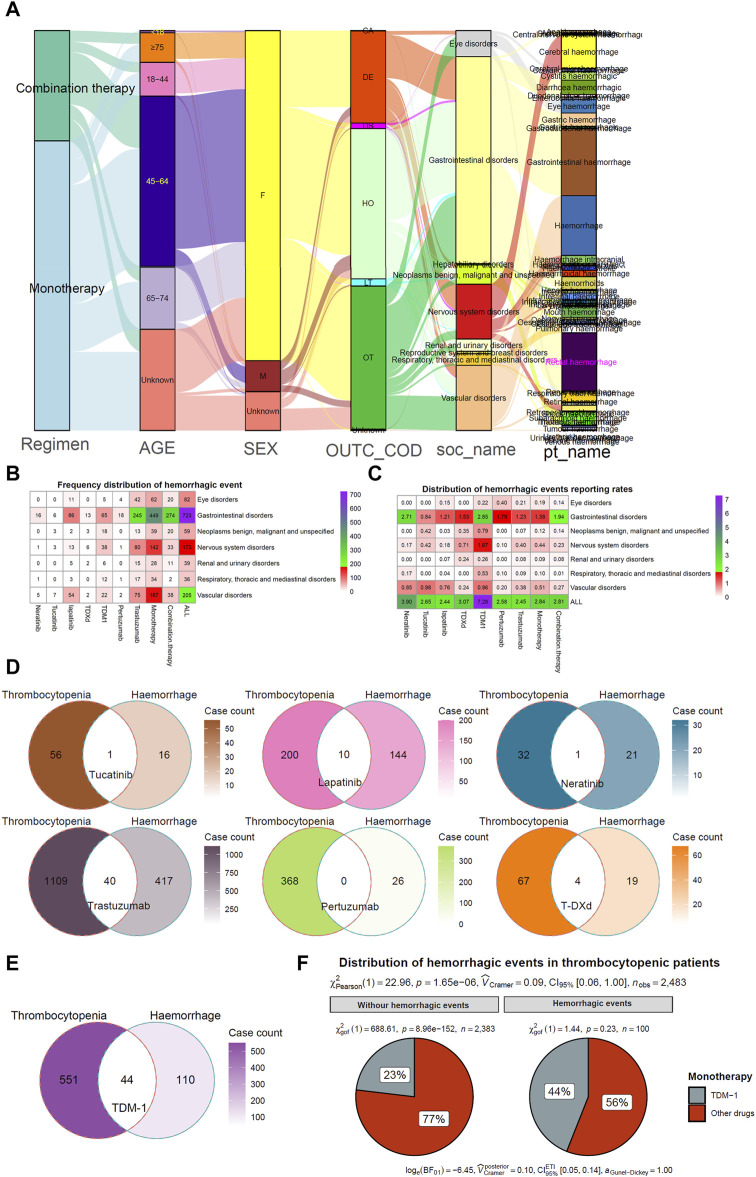
Hemorrhagic events and thrombocytopenia with HER2 inhibitors. **(A)** Mulberry map of clinical characteristics of patients with hemorrhagic events. Data were collected from regimen, age, sex, outcome (OUTC_COD), SOC types (soc_name), and preferred terms types (pt_name). Unknown indicates those not explicitly stated in the factor. **(B, C)** Heat map visualization of frequency distribution and reporting rates distribution of hemorrhagic events. **(D, E)** Wayne Map visualizes the overlapping relationship between haemorrhage and thrombocytopenia in patients receiving HER2 inhibitor therapy. **(F)** Chi-Square Test compares differences in Hemorrhagic events between TDM-1 and other HER2 inhibitors in patients reporting the presence of thrombocytopenia.

They were considering that hemorrhagic events, especially haemorrhage of vital organs, are severe or even lethal AEs. Hemorrhagic events associated with different HER2 inhibitors were further explored. The statistics on the percentage of the number of reported cases found the highest percentage of patients in the 45–64 age range (544 cases, 43%), followed by the 65–74 age range (198 cases, 16%) and the 18–44 age range (108 cases, 8.5%) (Figure 3A; Table 1). The overall percentage of patients who experienced a hemorrhagic event that resulted in death was 23% (294 cases). The top three frequencies of hemorrhagic events originating from gastrointestinal disorders (661 cases, 52%), vascular disorders (208 cases, 16%), and nervous system disorders (175 cases, 14%) (Figure 3B). Specifically, gastrointestinal haemorrhage (214 cases, 17%), rectal haemorrhage (186 cases, 15%), and cerebral haemorrhage (107 cases, 8.4%) occurred with relatively high frequency. In addition, TDM-1 (7.28%) had the highest total adverse event reporting rate of any drug (Figure 3C). However, the reported rate of hemorrhagic events in the combination therapy group (2.81%) was close to that of monotherapy (2.84%).

**TABLE 1:** Clinical characteristics of patients with hemorrhagic events.

Characteristic	N = 1,274^1^
Regimen	
Combination therapy	352 (28%)
Monotherapy	922 (72%)
Age	
<18	7 (0.5%)
≥75	94 (7.4%)
18–44	108 (8.5%)
45–64	544 (43%)
65–74	198 (16%)
Unknown	323 (25%)
Sex	
F	1,051 (82%)
M	99 (7.8%)
Unknown	124 (9.7%)
OUTC_COD	
CA	1 (<0.1%)
DE	294 (23%)
DS	18 (1.4%)
HO	477 (37%)
LT	23 (1.8%)
OT	457 (36%)
Unknown	4 (0.3%)
soc_name	
Eye disorders	83 (6.5%)
Gastrointestinal disorders	661 (52%)
Hepatobiliary disorders	4 (0.3%)
Neoplasms benign, malignant and unspecified	59 (4.6%)
Nervous system disorders	175 (14%)
Renal and urinary disorders	39 (3.1%)
Reproductive system and breast disorders	8 (0.6%)
Respiratory, thoracic and mediastinal disorders	37 (2.9%)
Vascular disorders	208 (16%)
pt_name	
Anal haemorrhage	7 (0.5%)
Arterial haemorrhage	1 (<0.1%)
Bronchial haemorrhage	3 (0.2%)
Central nervous system haemorrhage	3 (0.2%)
Cerebral haemorrhage	107 (8.4%)
Cerebral microhaemorrhage	1 (<0.1%)
Conjunctival haemorrhage	9 (0.7%)
Cystitis haemorrhagic	27 (2.1%)
Diarrhoea haemorrhagic	46 (3.6%)
Duodenal ulcer haemorrhage	8 (0.6%)
Enterocolitis haemorrhagic	7 (0.5%)
Eye haemorrhage	44 (3.5%)
Gastric haemorrhage	43 (3.4%)
Gastritis haemorrhagic	5 (0.4%)
Gastroduodenal haemorrhage	2 (0.2%)
Gastrointestinal haemorrhage	214 (17%)
Haemorrhage	191 (15%)
Haemorrhage intracranial	26 (2.0%)
Haemorrhage urinary tract	4 (0.3%)
Haemorrhagic ascites	2 (0.2%)
Haemorrhagic stroke	12 (0.9%)
Haemorrhoidal haemorrhage	21 (1.6%)
Haemorrhoids	41 (3.2%)
Hepatic haemorrhage	4 (0.3%)
Internal haemorrhage	7 (0.5%)
Intestinal haemorrhage	22 (1.7%)
Intra-abdominal haemorrhage	1 (<0.1%)
Intracranial tumour haemorrhage	10 (0.8%)
Intraventricular haemorrhage	6 (0.5%)
Laryngeal haemorrhage	1 (<0.1%)
Lip haemorrhage	3 (0.2%)
Mouth haemorrhage	35 (2.7%)
Naevus haemorrhage	6 (0.5%)
Oesophageal haemorrhage	5 (0.4%)
Oesophageal varices haemorrhage	9 (0.7%)
Oesophagitis haemorrhagic	1 (<0.1%)
Optic disc haemorrhage	1 (<0.1%)
Pulmonary haemorrhage	27 (2.1%)
Rectal haemorrhage	186 (15%)
Renal haemorrhage	1 (<0.1%)
Respiratory tract haemorrhage	5 (0.4%)
Retinal haemorrhage	29 (2.3%)
Retroperitoneal haemorrhage	3 (0.2%)
Shock haemorrhagic	7 (0.5%)
Subarachnoid haemorrhage	19 (1.5%)
Thalamus haemorrhage	1 (<0.1%)
Thoracic haemorrhage	1 (<0.1%)
Tumour haemorrhage	43 (3.4%)
Ureteric haemorrhage	1 (<0.1%)
Urethral haemorrhage	1 (<0.1%)
Urinary bladder haemorrhage	5 (0.4%)
Uterine haemorrhage	8 (0.6%)
Venous haemorrhage	2 (0.2%)

Previous studies have suggested that thrombocytopenia induced by antineoplastic therapy may be a risk factor for hemorrhagic events (Elting et al., 2001; Chari et al., 2019). Thus, the overlapping relationship between patients with thrombocytopenia and those with bleeding events was counted. Figure 3D demonstrates that none of the six HER2 inhibitor monotherapy users had more than 10% of concurrent thrombocytopenia and bleeding events. However, 40% (44/110) of TDM-1 applicants who experienced hemorrhagic events had coexisting thrombocytopenia (Figure 3E). Thus, TDM-1 had a significantly higher rate of thrombocytopenia with coexisting bleeding events compared to other drugs (*p* < 0.001) (Figure 3F).

### 3.4 Evaluation of intracranial haemorrhage events

**FIGURE 4:**
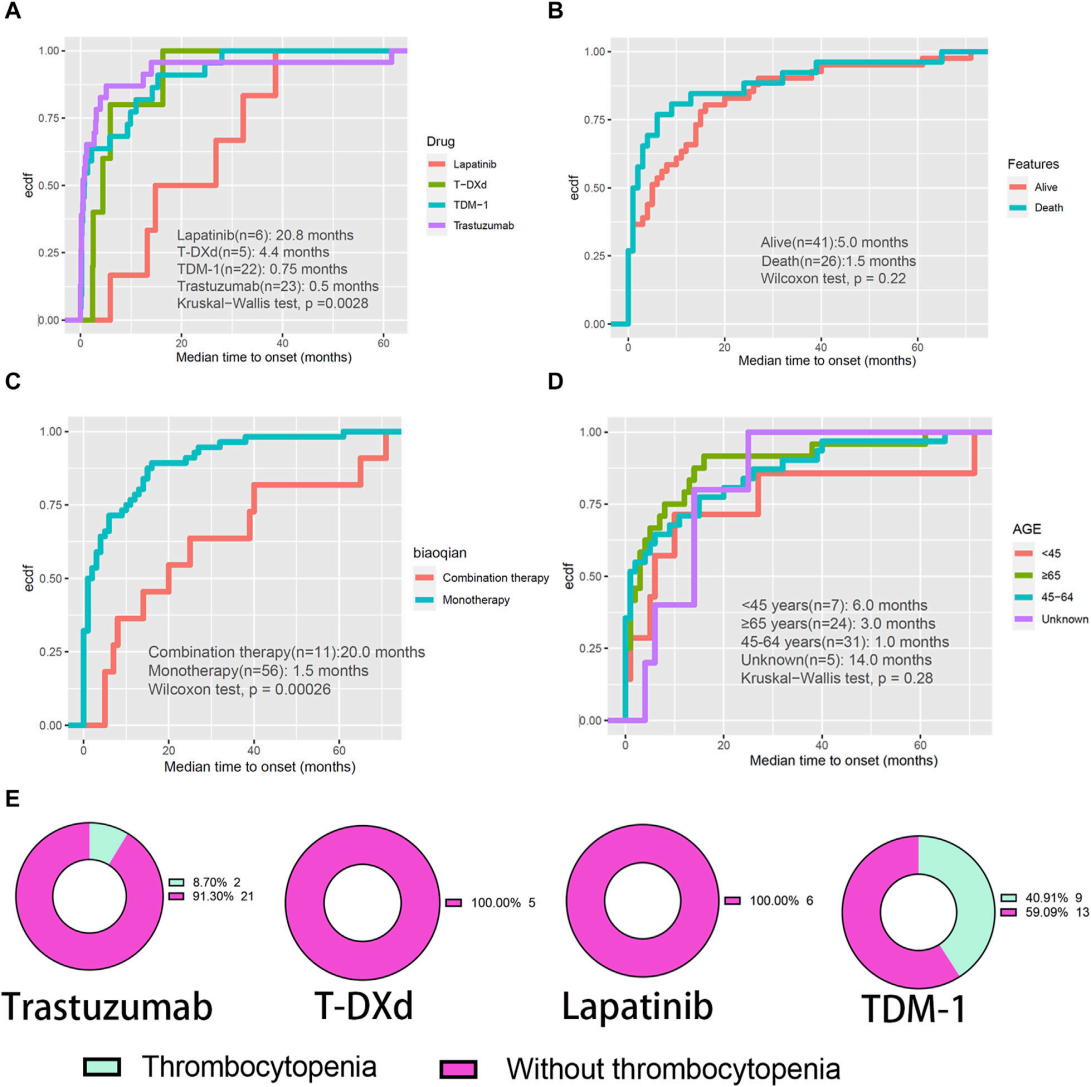
Evaluation of intracranial haemorrhage events. **(A–D)** The cumulative curve visualizes the interval between the initiation of HER2 inhibitors and the occurrence of intracranial haemorrhage. **(E)** Percentage of patients treated with four HER2 monotherapies who developed intracranial haemorrhage who had concomitant thrombocytopenia.

Intracranial haemorrhage is a severe and lethal adverse event. Evaluation of the interval between the initiation of HER2 inhibitors and the occurrence of intracranial haemorrhage is warranted. Among them, the median time to onset of intracranial haemorrhage was significantly higher for lapatinib than for trastuzumab, TDM-1, or T-DXd (*p* < 0.05) (Figure 4A). In addition, the median time to onset intracranial haemorrhage was significantly higher for combination therapy than for monotherapy (20.0 months VS. 1.5 months, *p* < 0.001) (Figure 4C). However, there was no significant difference in median time to onset intracranial haemorrhage between patients in different age groups or with different outcomes (Figures 4B, D). In addition, the distribution of thrombocytopenia in patients who developed intracranial haemorrhage was counted. The results again suggested that TDM-1 applicants who suffered intracranial haemorrhage were more likely to have thrombocytopenia complications (Figure 4E). It may be necessary to analyze further whether patients treated with TDM-1 are more likely to report nervous system disorders than other HER2 inhibitors.

### 3.5 AEs involving nervous system disorders

**FIGURE 5:**
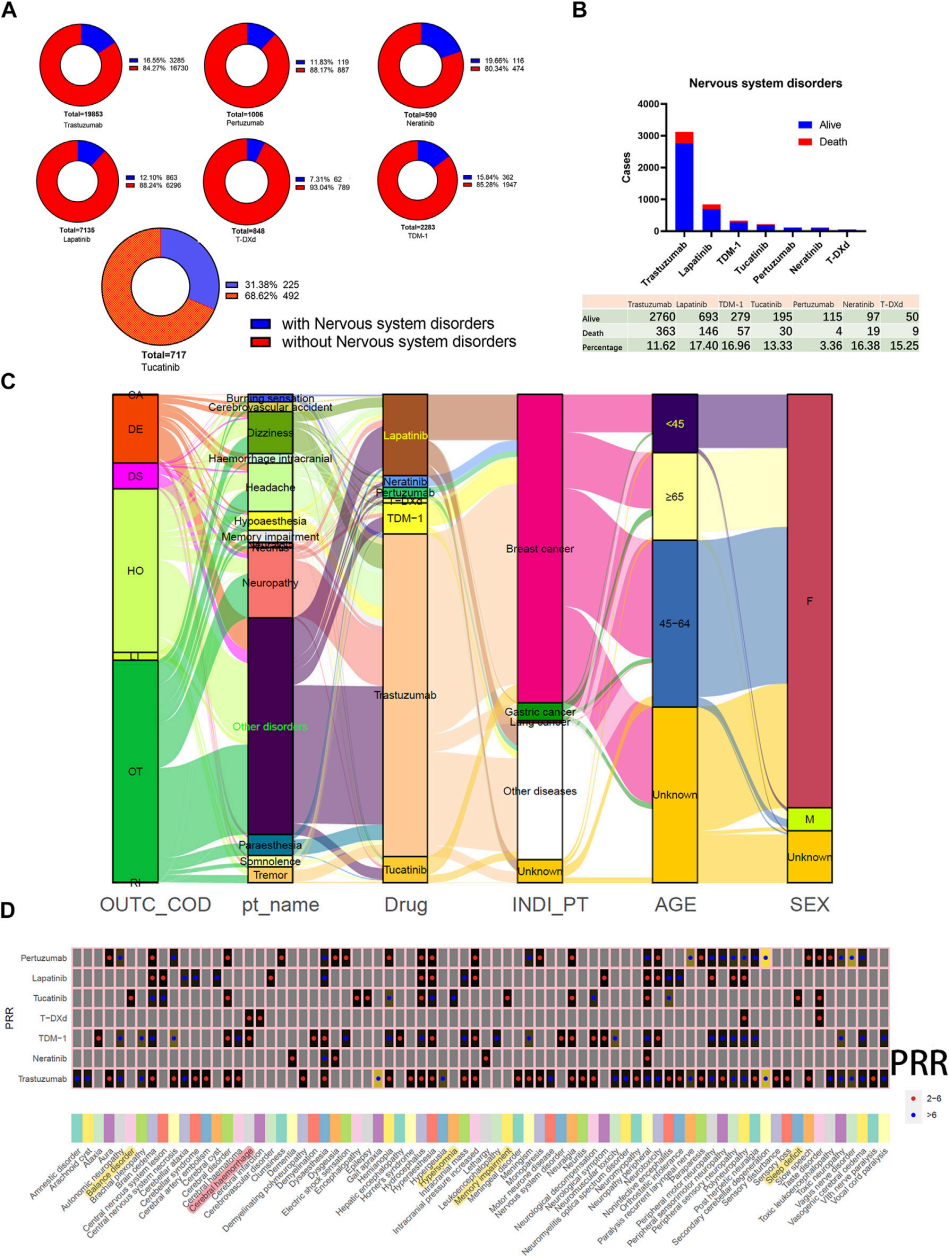
Adverse events involving nervous system disorders. **(A)** Distribution of the total number and percentage of patients with concomitant nervous system disorders in reports of HER2 monotherapy. **(B)** Bar graph statistics of the proportion and number of cases of living and dead patients presenting with nervous system disorders, and those not explicitly labeled in the outcome list have been excluded. **(C)** Mulberry map of clinical characteristics of patients with nervous system disorders. **(D)** Comparison of positive signal with nervous system disorders obtained by different HER2 inhibitors based on the Proportional Reporting Ratio (PRR) method. Red dots indicate PRR values in the 2.0–6.0 range. Blue dots indicate PRR values above 5.0.

Evaluating AEs involving nervous system disorders originating from HER2 inhibitors is becoming increasingly important as more and more drugs that enhance the control of intracranial lesions are developed. Of these, the highest percentage of patients treated with tucatinib (31.38%) developed nervous system disorders, and the lowest was with T-DXd (7.31%), and the percentage of TDM-1 was only 15.84% (Figure 5A). Figure 5B demonstrates the difference in the number of cases of AEs involving nervous system disorders reported by users of monotherapy with seven different HER2 inhibitors. The percentage of patients whose outcome was death fluctuated from 3.36% to 17.4% of the total. Neuropathy, headache, dizziness, paraesthesia, and memory impairment are common PTs (Figure 5C). The results of the disproportionality analysis suggested that cerebral haemorrhage is a positive signal for TDM-1 and T-DXd (Figure 5D). In addition, among the seven HER2 inhibitors, demyelination and leukoencephalopathy were the only positive signals in TDM-1 applicants. Therefore, myelin damage in TDM-1 applicants might be worth emphasizing.

On the other hand, applicants of tucatinib had the highest reported rates of nervous system disorders, so the positive signals unique to this drug were also spotlighted. PPR signals were positive for encephalopathy, epilepsy, balance disorder, hypersomnia, memory impairment, and sleep deficit. In addition, neuropathy peripheral was also a factor in the high prevalence of tucatinib applicants. The number of reports of neuropathy peripheral (95 cases), memory impairment (83 cases), and balance disorder (37 cases) were among the top three PTs with PRR-positive signals for tucatinib. Since that memory impairment is a relatively insidious symptom, it may be worth further attention.

## 4 Discussion

Reducing the risk of various AEs associated with HER2 inhibitors and improving patients’ quality of life is one of the keys to the success of antitumor therapy. The FAERS database provides us with follow-up data on drug safety, allowing us to better monitor the distribution of AEs for various drugs, especially those that occur outside the hospital. By comparing eight HER2 inhibitors horizontally and vertically, our study partially demonstrates the changes and status of anti-HER2 therapy over the past decade or so. We visualized the number of various HER2 inhibitors applied alone, sequentially, or combined based on overlap relationships (Figure 1A). Our statistics suggest that trastuzumab remains the most reported drug for AEs, both in number and in combination. However, more than 300 tumor patients had been treated with four or even five HER2 inhibitors, which predicts fair compatibility between these drugs. These drug combinations will continue to evolve as more emerging HER2 inhibitors advance in therapeutic lines and efficacy is confirmed (Giordano et al., 2023; Najminejad et al., 2023).

The distribution of adverse event reporting rates suggests that those patients treated with two or more HER2 inhibitors were more likely to report AEs for SOCs such as blood and lymphatic system disorders, infections and infestations, investigations, and neoplasms benign, malignant, and unspecified compared to monotherapy. This phenomenon may contribute to may be the accumulation of HER2 inhibitor-related toxicity, disease progression, and the superimposition of toxicity from other antitumor agents, among other factors. In addition, the reported rates of AEs demonstrated that TDM-1 and T-DXd appeared significantly different in multiple SOCs (Figure 1C). For example, patients treated with T-DXd reported a higher proportion of respiratory, thoracic, and mediastinal disorders, whereas patients treated with TDM-1 reported a higher proportion of hepatobiliary disorders. These results are consistent with previous studies (Yan et al., 2016; Battisti et al., 2020; Swain et al., 2022). In addition, we visualized the distribution of all reported AEs in patients treated with only one HER2 inhibitor by mulberry plots, incorporating seven inhibitors in addition to pyrotinib (Figure 2B, Supplementary Figure S1). These data may help clinicians or patients choose appropriate anti-HER2 therapy. Indeed, our study once again demonstrates the tendency of small-molecule drugs to be highly prevalent in gastrointestinal disorders, especially such common disorders as diarrhea, nausea, and vomiting. Therefore, good management of gastrointestinal AEs is necessary when applying this class of drugs.

The correlation between the occurrence of hemorrhagic events during antitumor therapy and thrombocytopenia has remained inconclusive (Wuerstlein et al., 2022; Wang et al., 2023). Considering the dangers of hemorrhage, especially the fatal consequences of intracranial and gastrointestinal hemorrhage, our study focused on hemorrhagic events potentially associated with anti-HER2 therapy. Few previous studies have conducted side-by-side comparisons of these drugs. We found that TDM-1 had a significantly higher proportion of reported hemorrhagic events than other HER2 inhibitors. Furthermore, patients treated with TDM-1 who experienced hemorrhagic events had a significantly higher rate of thrombocytopenia. Based on the inherent flaws in the evidence from the spontaneous reporting system, we cannot directly assume that thrombocytopenia-induced hemorrhagic events in patients treated with TDM-1, especially since 60% (66/110) of such patients did not report the occurrence of thrombocytopenia (Figure 3E). However, thrombocytopenia may have partially influenced the occurrence of haemorrhage. Of course, we also noted that the percentage of bleeding events intersecting with thrombocytopenia was less than 10% for each of the other six HER2 inhibitors, which may prove that thrombocytopenia is not strongly correlated with bleeding events in users of these drugs. In addition, our study also attempted to investigate the effect of different factors on the time of initiation of intracranial hemorrhagic events. Age and outcome of AEs are not important intervening factors. We note that the median time to onset of intracranial haemorrhage associated with trastuzumab (0.5 months) and TDM-1 (0.75 months) was short. This result warns us that we need to be concerned about the possibility of hemorrhagic events when applying these HER2 inhibitors initially.

As the control of extracranial diseases improves, the probability of malignant tumors metastasizing within the central nervous system gradually increases (Ferrario et al., 2022). The blood-brain barrier is a major obstacle to the effective penetration and diffusion of antitumor drugs to intracranial lesions (Giordano et al., 2023). Therefore, some HER2 inhibitors, such as tucatinib and neratinib, emphasize high blood-brain barrier permeability (Giordano et al., 2023). However, could increase intracranial drug concentrations exacerbate or induce some neurologic toxicity that was previously easily overlooked in the clinic? Our study found that the reported rates of nervous system disorders associated with either tucatinib or neratinib were more than twice that of other HER2 inhibitors (Figure 1C). The FAERS database has reported 83 cases of memory impairment in patients treated with tucatinib. This is rarely mentioned in previous studies.

### 4.1 Limitation

The present study is mainly based on the exploration of public databases, but further validation of our analytical conclusions requires more real-world prospective studies and large-scale clinical trials. Due to the limitations of spontaneous reporting systems and the differences in the importance placed on AEs in different countries and regions, there are situations such as under-reporting, missing patient basic information, and inability to estimate the incidence of ADR. The AEs mining method used in this paper is the disproportionality analysis, which highlights the differences in the number of reports between a specific drug-event combination in the database and other drug-event combinations in the database, and is only used to generate drug safety signals (i.e., provide clues to drug safety), and cannot prove the causal relationship between drug events.

### 4.2 Conclusion

The types and reporting rates of AEs associated with different HER2 inhibitor treatments vary across multiple systems. In addition, hemorrhagic events concomitant with TDM-1 treatment and nervous system disorders concomitant with tucatinib treatment may be worthy of attention.

## Data Availability

The original contributions presented in the study are included in the article/Supplementary Material, further inquiries can be directed to the corresponding authors.

## Abbreviations

BC, Breast Cancer; HER2, Human Epidermal Growth Factor Receptor 2; ADC, Anti-body Drug Conjugates; AEs, Adverse Events; FAERS, The US Food and Drug Administration Adverse Event Reporting System; PRR, Proportional Reporting Ratio; PTs, Preferred terms; SOCs, System Organ Classes; TDM-1, Trastuzumab emtansine; T-DXd, Trastuzumab deruxtecan.
