# Supplementary material for: Comparing the difference of adverse events with HER2 inhibitors: a study of the FDA adverse event reporting system (FAERS)
Source: Front Pharmacol. 2024 Jan 24;15:1288362. doi: 10.3389/fphar.2024.1288362 (PMC10847310; doi:10.3389/fphar.2024.1288362)
Supplement: Supplementary file 1 [file DataSheet1.pdf]

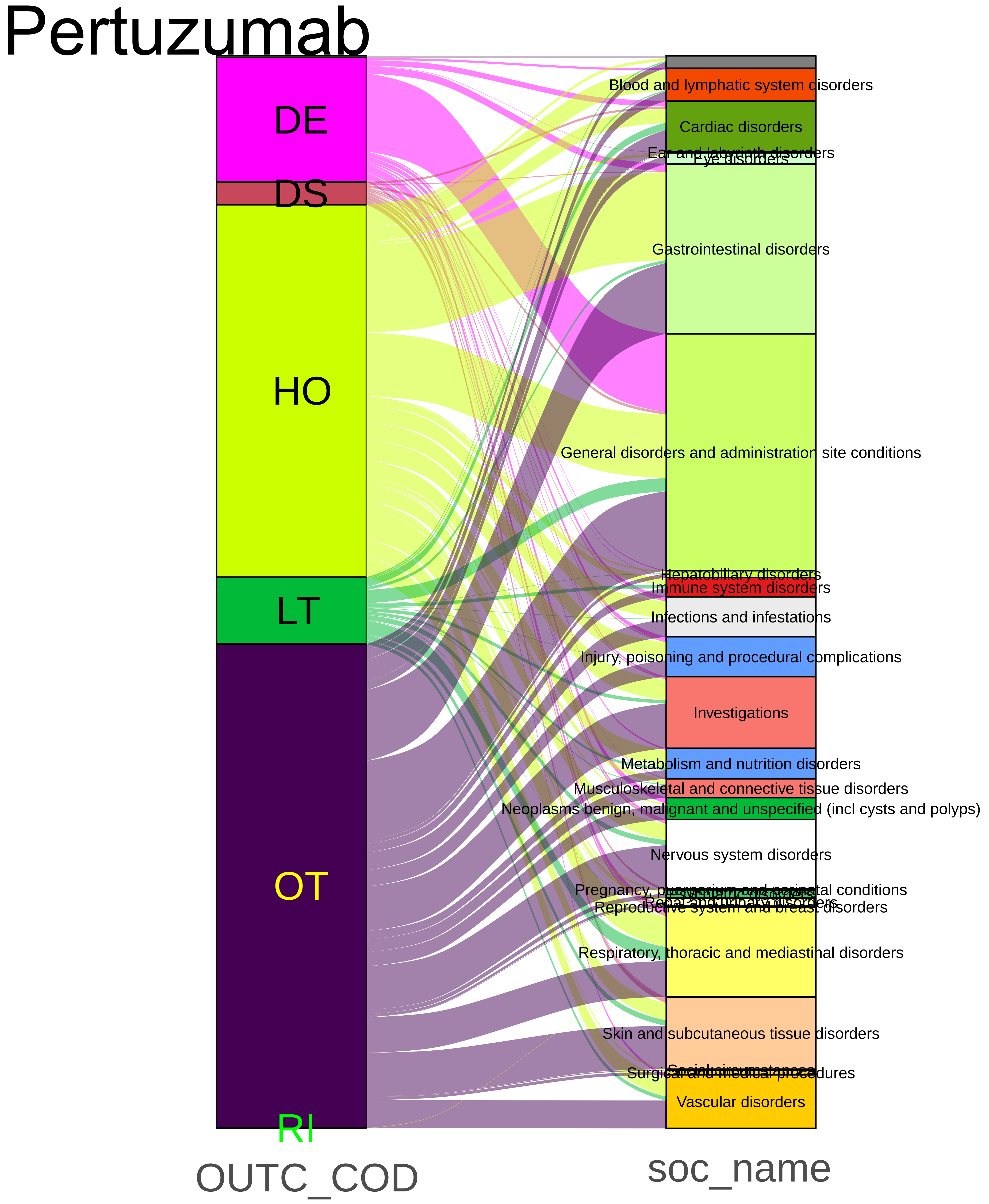
Pertuzumab
DE
DS
HO
LT
OT
RI
OUTC_COD
soc_name
Blood and lymphatic system disorders
Cardiac disorders
Ear and labyrinth disorders
Eye disorders
Gastrointestinal disorders
General disorders and administration site conditions
Hepatobiliary disorders
Immune system disorders
Infections and infestations
Injury, poisoning and procedural complications
Investigations
Metabolism and nutrition disorders
Musculoskeletal and connective tissue disorders
Neoplasms benign, malignant and unspecified (incl cysts and polyps)
Nervous system disorders
Pregnancy, puerperium and perinatal conditions
Psychiatric disorders
Renal and urinary disorders
Reproductive system and breast disorders
Respiratory, thoracic and mediastinal disorders
Skin and subcutaneous tissue disorders
Social circumstances
Surgical and medical procedures
Vascular disorders

TDM-1

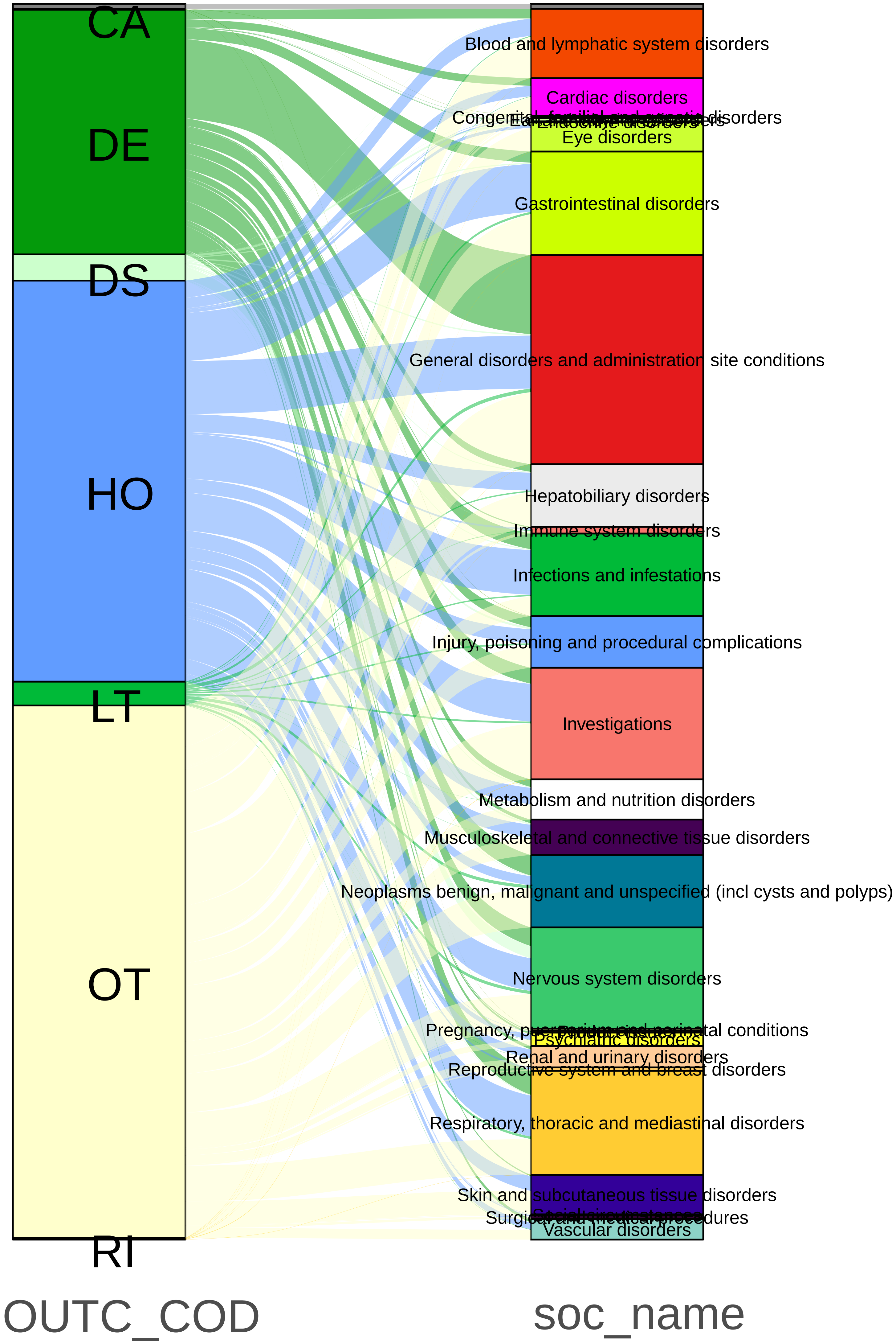
CA
DE
DS
HO
LT
OT
RI
OUTC_COD
soc_name
Blood and lymphatic system disorders
Cardiac disorders
Eye disorders
Gastrointestinal disorders
General disorders and administration site conditions
Hepatobiliary disorders
Immune system disorders
Infections and infestations
Injury, poisoning and procedural complications
Investigations
Metabolism and nutrition disorders
Musculoskeletal and connective tissue disorders
Neoplasms benign, malignant and unspecified (incl cysts and polyps)
Nervous system disorders
Renal and urinary disorders
Reproductive system and breast disorders
Respiratory, thoracic and mediastinal disorders
Skin and subcutaneous tissue disorders
Vascular disorders

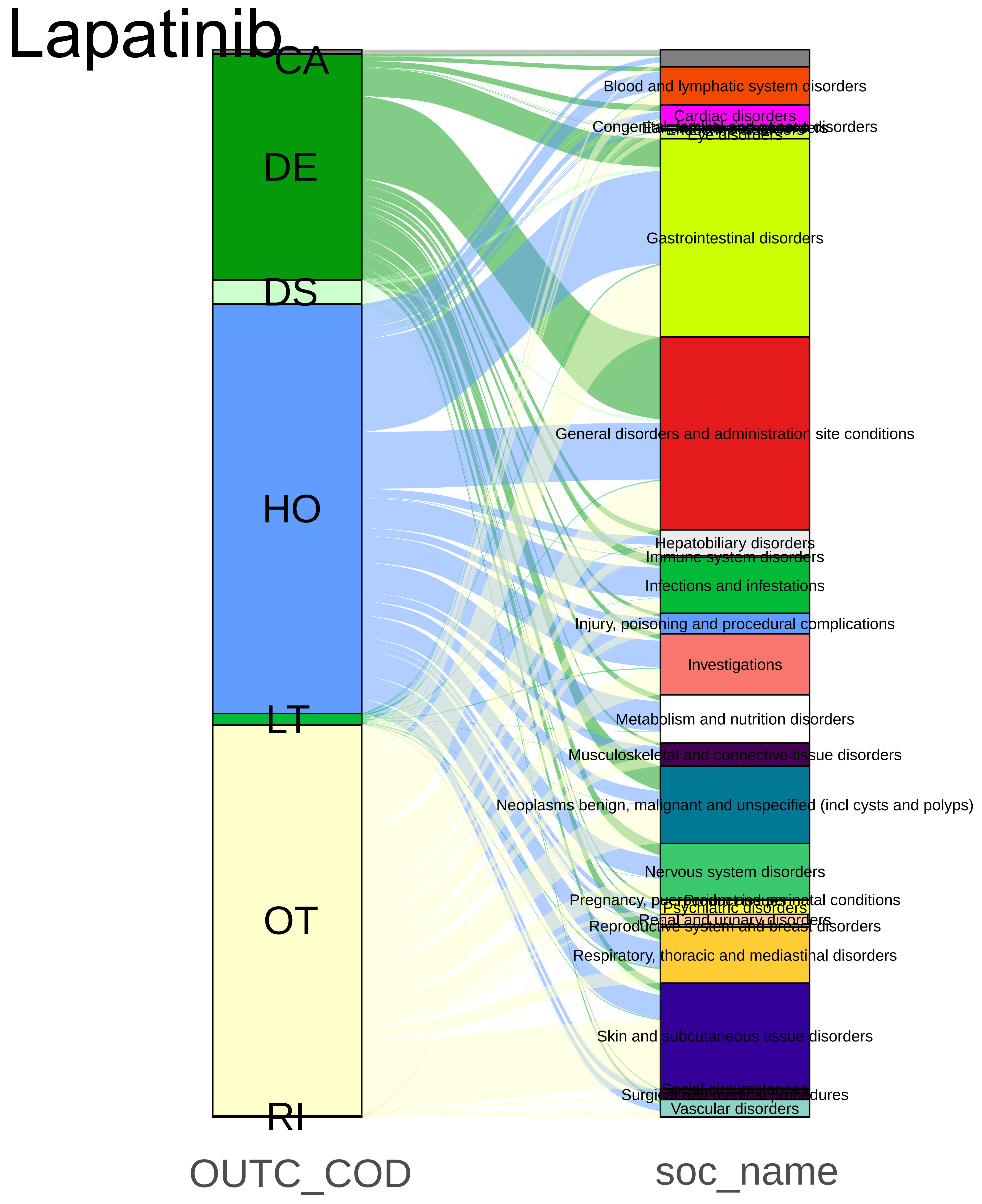
Lapatinib
CA
DE
DS
HO
LT
OT
RI
Blood and lymphatic system disorders
Cardiac disorders
Eye disorders
Gastrointestinal disorders
General disorders and administration site conditions
Hepatobiliary disorders
Immune system disorders
Infections and infestations
Injury, poisoning and procedural complications
Investigations
Metabolism and nutrition disorders
Musculoskeletal and connective tissue disorders
Neoplasms benign, malignant and unspecified (incl cysts and polyps)
Nervous system disorders
Psychiatric disorders
Renal and urinary disorders
Reproductive system and breast disorders
Respiratory, thoracic and mediastinal disorders
Skin and subcutaneous tissue disorders
Surgical and medical procedures
Vascular disorders
OUTC_COD
soc_name

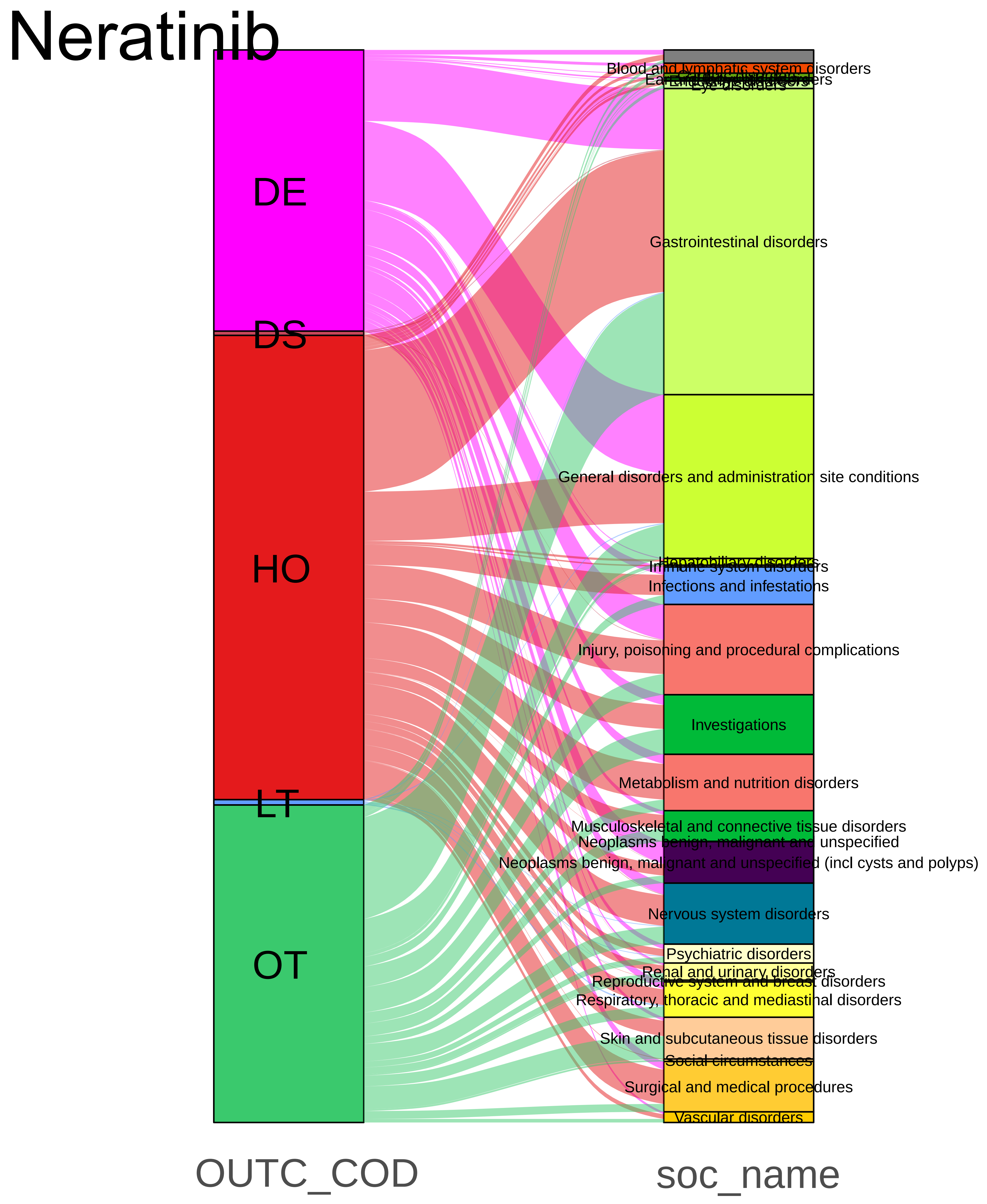

Neratinib
DE
DS
HO
LT
OT
Blood and lymphatic system disorders
Eye disorders
Gastrointestinal disorders
General disorders and administration site conditions
Hepatobiliary disorders
Immune system disorders
Infections and infestations
Injury, poisoning and procedural complications
Investigations
Metabolism and nutrition disorders
Musculoskeletal and connective tissue disorders
Neoplasms benign, malignant and unspecified
Neoplasms benign, malignant and unspecified (incl cysts and polyps)
Nervous system disorders
Psychiatric disorders
Renal and urinary disorders
Reproductive system and breast disorders
Respiratory, thoracic and mediastinal disorders
Skin and subcutaneous tissue disorders
Social circumstances
Surgical and medical procedures
Vascular disorders
OUTC_COD
soc_name

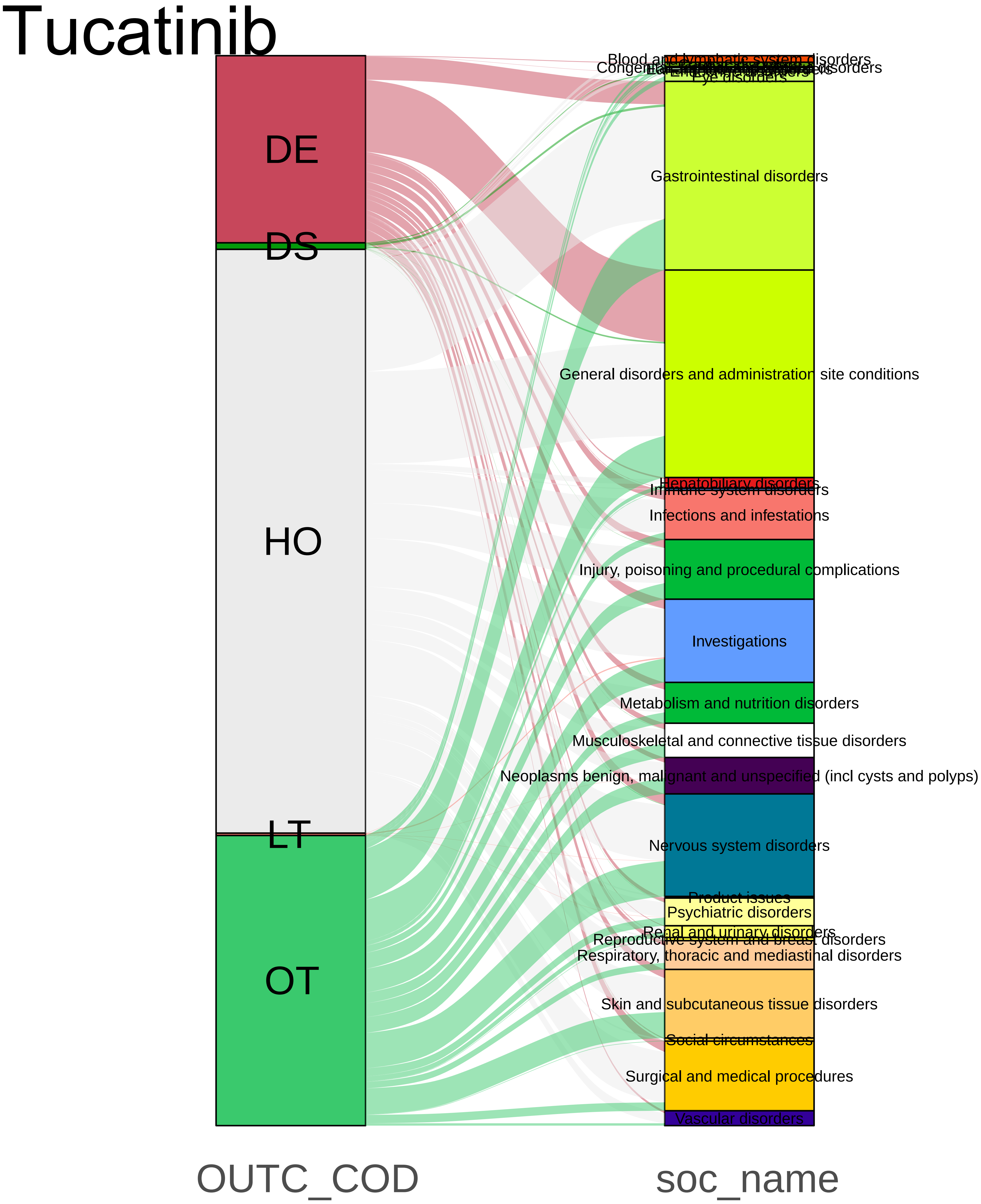
Tucatinib
DE
DS
HO
LT
OT
Blood and lymphatic system disorders
Eye disorders
Gastrointestinal disorders
General disorders and administration site conditions
Hepatobiliary disorders
Immune system disorders
Infections and infestations
Injury, poisoning and procedural complications
Investigations
Metabolism and nutrition disorders
Musculoskeletal and connective tissue disorders
Neoplasms benign, malignant and unspecified (incl cysts and polyps)
Nervous system disorders
Product issues
Psychiatric disorders
Renal and urinary disorders
Reproductive system and breast disorders
Respiratory, thoracic and mediastinal disorders
Skin and subcutaneous tissue disorders
Social circumstances
Surgical and medical procedures
Vascular disorders
OUTC_COD
soc_name

Comparing The Difference of Adverse Events with HER2 Inhibitors: A Study of The FDA Adverse Event Reporting System (FAERS)

Figure Legend

Supplementary Figure 1. Mulberry diagram visualizing the correspondence of adverse events (soc_name) and outcomes (OUTC_COD) reported in cases treated with pertuzumab, TDM-1, lapatinib, neratinib, or tucatinib.
